# Surgical Safety Checklists in Children’s Surgery: Surgeons’ Attitudes and Review of the Literature

**DOI:** 10.1097/pq9.0000000000000108

**Published:** 2018-10-16

**Authors:** Jessica Roybal, KuoJen Tsao, Shawn Rangel, Madelene Ottosen, David Skarda, Loren Berman

**Affiliations:** From the * Department of Pediatric Surgery, Ochsner Hospital for Children, New Orleans, LA.; † Department of Pediatric Surgery, McGovern Medical School at The University of Texas Health Science Center at Houston, TX; ‡ Department of Surgery, Boston Children’s Hospital, Harvard Medical School, Boston, MA; ¶ University of Texas Houston-Memorial Hermann Center for Healthcare Quality and Safety, Houston, TX; ‖ Department of Pediatric Surgery, University of Utah, Salt Lake City, UT; ** Department of Pediatric Surgery, Nemours/Alfred I. duPont Hospital for Children, Wilmington, DE; †† Sidney Kimmel College at Thomas Jefferson University, Philadelphia, PA.

## Abstract

**Introduction:**

Surgical safety checklists (SSCs) aim to create a safe operating room environment for surgical patients. Provider attitudes toward checklists affect their ability to prevent harm. Pediatric surgeons’ perceptions surrounding SSCs, and their role in improving patient safety, are unknown.

**Methods:**

American Pediatric Surgical Association members conducted an online survey to evaluate the use of and attitudes toward SSCs. The survey measured surgeons’ perceptions of checklists, including the components that make them effective and barriers to participation. To better evaluate the available data on SSCs, the authors performed a systematic literature review on the use of SSCs with a focus on pediatric studies.

**Results:**

Of the 353 survey respondents, 93.6% use SSCs and 62.6% would want one used in their own child’s operation, but only 54.7% felt that checklists improve patient safety. Reasons for checklist skepticism included the length of the checklist process, a distraction from thoughtful patient care, and lack of data supporting use. Literature review shows that checklists improve communication, promote teamwork, and identify errors, but do not necessarily decrease morbidity. Staff perception is a major barrier to implementation.

**Conclusions:**

Almost all pediatric surgeons participate in SSCs at their institutions, but many question their benefit. Better pediatric surgeon engagement in checklist use is needed to change the safety culture, improve operating room communication, and prevent harm.

## Abstract 2

Supplemental Digital Content is available in the text.

## INTRODUCTION

Since the 1930s, checklists have been used in aviation to prevent accidents due to human error.^1^ In 2008, the World Health Organization (WHO) launched the “Safe Surgery Saves Lives” campaign and developed a Surgical Safety Checklist (SSC);^2,3^ various forms have been adopted throughout the world. After implementation of the WHO’s 19-item surgical checklist in 8 different hospitals, the death rate decreased from 1.5% to 0.8%, and the complication rate decreased from 11.0% to 7.0%.^4^ On the basis of an annual number of 234 million operations performed each year globally, the WHO estimates that effective implementation of the WHO SSC could prevent at least half a million deaths per year worldwide.^2,4^ In the United States, since calendar year 2015, the Centers for Medicare and Medicaid Services has made “safe surgery checklist use” one of the measures in the Ambulatory Surgical Center Quality Reporting payment program.^5^ With the WHO SSC as a guideline, institutions throughout the world are adopting their versions of the surgical checklist with opportunities for a pause before induction of anesthesia, before incision, and at the end of the case.^6^

Prevention of death and complications is a clear goal of SSCs, but it is not the only goal. Checklists have additional benefits that indirectly affect patient care. SSCs are shown to improve communication^7–9^ and teamwork^8,10,11^ and reduce observable errors relating to poor team skills.^10^ However, not all studies have found checklists to be effective. The checklist-based quality improvement program in Michigan, known as Keystone Surgery, did not improve surgical complications or 30-day mortality rates.^12^ Successful implementation of an SSC depends on individual “buy-in” and surgeon engagement in the process.^11,13^ Most institutions use a checklist because it is a safety measure; however, meaningful use of the checklist is highly variable. Furthermore, checklist adoption, acceptance, and adherence may differ between surgical subspecialties and between surgeons caring for adults versus children. Checklists are not always designed for pediatric patients and may have irrelevant components if used for both adult and pediatric patients. Workflow can be negatively affected by an irrelevant checklist. Therefore, a positive perception of the SSC is paramount to its success.

Since the hallmark WHO report,^4^ the majority of studies show that checklists improve patient safety and outcomes in adults. However, researchers have not adequately investigated the effect of SSCs in the pediatric population. The current investigators designed a survey to assess safety knowledge, attitudes, and perceptions of North American pediatric surgeons and to specifically gauge the “buy-in” of the American Pediatric Surgical Association (APSA) membership on checklists. To support understanding of surgical checklists, the investigators conducted a literature review aiming to summarize the current data on the utility of SSCs in both adult and pediatric surgery.

## METHODS

### Study Design

All active APSA members were e-mailed an invitation asking them to participate in a survey on SurveyMonkey. The survey was developed by the APSA Quality and Safety Committee to measure patient safety attitudes and perceptions of the membership. It collected demographic information and practice setting and asked surgeons whether they functioned in any formal leadership, education, or safety roles within their hospital. This report focuses on checklist-specific survey content. Questions were designed to quantify surgeon participation in the preinduction, preincision, and postoperative debriefing checklists and measure surgeons’ attitudes about the effectiveness of checklists using Likert scales. Respondents had the opportunity to elaborate on certain survey questions with open-ended responses. APSA members were encouraged to participate in the survey with 2 follow-up reminder e-mails over a 3-week interval.

### Variables and Data Analysis

The current investigators performed standard frequency analyses for surgeons’ responses regarding whether checklists exist in their hospital and to summarize surgeons’ beliefs about whether checklists improve patient safety. They tested associations between surgeon characteristics (years since fellowship, practice setting, leadership/safety/educational roles) and attitudes toward the SSC using a chi-square test for categorical variables and Kruskal-Wallis test for continuous variables. Likert scale responses were dichotomized into agree/strongly agree versus neutral/disagree/strongly disagree. A *P* value < 0.05 was statistically significant.

The authors analyzed open-ended response questions using content analysis to enhance understanding of quantitative findings. The content analysis method is a systematic data coding and analysis procedure^14–16^ that allocates specific quotes from respondents into a code structure that is developed through an iterative process. The codes are then used to group the data into representative themes. The investigators selected specific quotes to illustrate the broader themes, and these are noted in italics. Four separate reviewers (including an expert in qualitative research) evaluated the open-ended responses and formed consensus on the themes that emerged.

### Literature Review

In addition to the survey, we conducted a literature search to identify systematic reviews of safety checklists in surgery and studies focusing on checklists in the pediatric surgical population. The online databases Medline, Embase, and PubMed were searched using the terms “surgical checklists” AND “systematic review,” “safety checklists” AND “systematic review,” “surgical safety checklists” AND “systematic review,” “pediatric surgery,” “checklist,” AND “systematic review,” and “children’s surgery,” “checklist,” AND “systematic review.” Studies were limited to those that focused on surgery/perioperative checklists, were written in the English language, and were peer-reviewed and included an abstract.

## RESULTS

### Demographics and Other Respondent Characteristics

A total of 928 APSA members received the survey, and 353 responded (38% response rate). The majority of respondents operate primarily within a children’s hospital, 49.7% freestanding and 38.1% within an adult medical center. Most respondents were in an academic (65.3%) or mixed (25.3%) practice, with only 9.4% in private practice. The majority of respondents (56.7%) self-reported holding leadership positions at their institutions (eg, chair or division chief), with 43.4% in education and 21.0% in safety positions. The median number of years since completion of pediatric surgery fellowship was 13 (range, 0–32 years).

### Practice and Attitudes Surrounding SSC

Most respondents (93.6%) reported compliance with SSCs at their institution, but only 54.7% felt that checklists improve patient safety. Respondents most consistently reported doing the preincision checklist (83.8% of respondents stated that they do it all of the time), followed by the preinduction checklist (79%), and then the postoperative debriefing checklist (29%). The majority of respondents (54.5%) agreed strongly that the checklists improve operating room communication, but only 35.6% agreed that they prevent harm and 24.1% that they benefit every case. Only 62.6% agreed strongly that they would want it used in their own child’s operation. Being in a safety position was the only respondent characteristic correlated with believing that checklists improve patient safety or wanting the checklist used in one’s own child’s operation (Table 1).

**Table 1.**
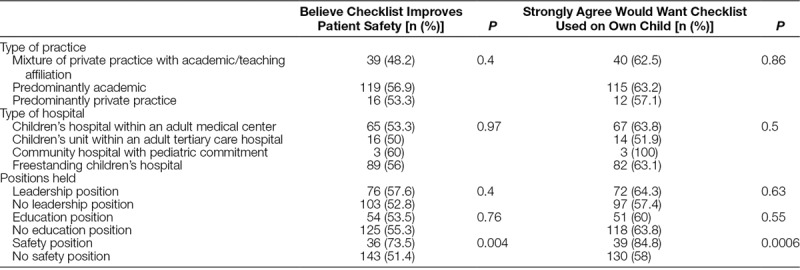
Associations between Surgeon Characteristics and Attitudes toward Surgical Safety Checklists

In the open-ended questions, several themes emerged to explain a lack of enthusiasm for SSCs/handoffs:

1) Checklists are too long and contain information that is not relevant to the current case. Three respondents had these comments:

“Checklists only work if they are short. So many checklists are filled with useless info. As a pilot, I use a checklist when I fly. It encourages safety by being short, simple, and to the point.”

“There are generic elements to all surgical procedures that have some applications in the generic checklists currently available. What would be far more effective would be computer generated checklists SPECIFIC for the procedure scheduled.”

“Safety checklist has become too encompassing beyond safety issues so that safety factors are getting lost in the long checklist, which has become another form to complete.”

2) Checklists result in mindlessness as opposed to mindfulness and can distract from thoughtful patient care as described by these 4 respondents:

“All this devotion to checklists and process has become a form of fetishism, and often is a distraction from the task at hand.”

“The surgical checklist is highly ineffective in modern Western hospitals. This was shown, but ignored, in the original paper. It is a meme, a trend, and a fad. It does not actually improve real mindfulness, and often distracts from it.”

“People sometimes go through the motions but don’t really pay attention.”

“Like any checklist, it is used with varying degrees of a mechanical/begrudging fashion.”

3) Surgeons want to see data proving that checklists prevent adverse events. As 2 surgeons stated:

“There may be one case in 10,000 in which it makes a difference. I personally haven’t seen it.”

“Of the tools that are available, none have led to new systems that demonstrably decrease error or improve safety.”

In contrast to the negative comments summarized above, some surgeons saw some utility in the checklist depending on the situation.

“It was my practice to discuss the more complicated cases at the beginning of the day to identify the need for additional anesthesia/nursing/surgical care or unusual equipment. Current safety lists may have influenced some groups to use a similar practice. However, for most routine cases, the safety lists (required for all cases) add little or nothing to the case or safety of the patient.”

“It has improved patient safety in SOME cases.”

Many other respondents voiced strong support for SSCs as illustrated by these 3 respondents:

“SSC has definitely improved OR [operating room] safety. NO wrong side procedures, NO wrong procedures since it was introduced and adopted. Also caught several ‘near misses,’ also improved timeliness of preop antibiotics.”

“I have been trying for years to implement a safety checklist.”

“It has become hard-wired and is an improvement.”

### Literature Review

The investigators identified 34 manuscripts, of which 11 met inclusion criteria and were reviewed in detail. Only 2 studies address pediatric surgery patients,^17,18^ and the remainder focus on the adult literature. The first pediatric surgery study, by MacDonald and Sevdalis,^17^ systematically reviews 20 studies of patient safety interventions including the SSC. The authors note that pediatric cardiothoracic surgery account for the majority of the children’s safety literature. Fifteen studies (75%) use a checklist tool as part of their safety intervention; however, only 5 studies use checklists as their primary safety intervention. The 5 checklist-oriented studies evaluate checklist adherence and perioperative patient safety, but none assess the rate of complications, and only 1 addresses the near misses as a result of checklist adherence.

The second pediatric surgery study, a systematic review, by Lagoo and colleagues,^18^ evaluates the effectiveness and meaningful use of pediatric SSCs and their implementation strategies. The authors include 26 studies, all of which are cross-sectional or cohort studies. They rate 6 of them as poor overall. Of the 20 studies rated fair or good, only 16 evaluate SSCs in the operating room or perioperative setting. Three studies measure the effectiveness of checklists on morbidity and mortality with mixed results; one of these is primarily in adults with a small pediatric component. Four studies assess parental involvement in SSCs. Nine studies evaluate compliance with checklists, 11 studies investigate the implementation of checklists, and 8 studies assess attitudes related to checklist use and the effect of SSCs on safety culture. Table 2 summarizes the results of the pediatric surgery-specific checklist studies written in the English language.

**Table 2.**
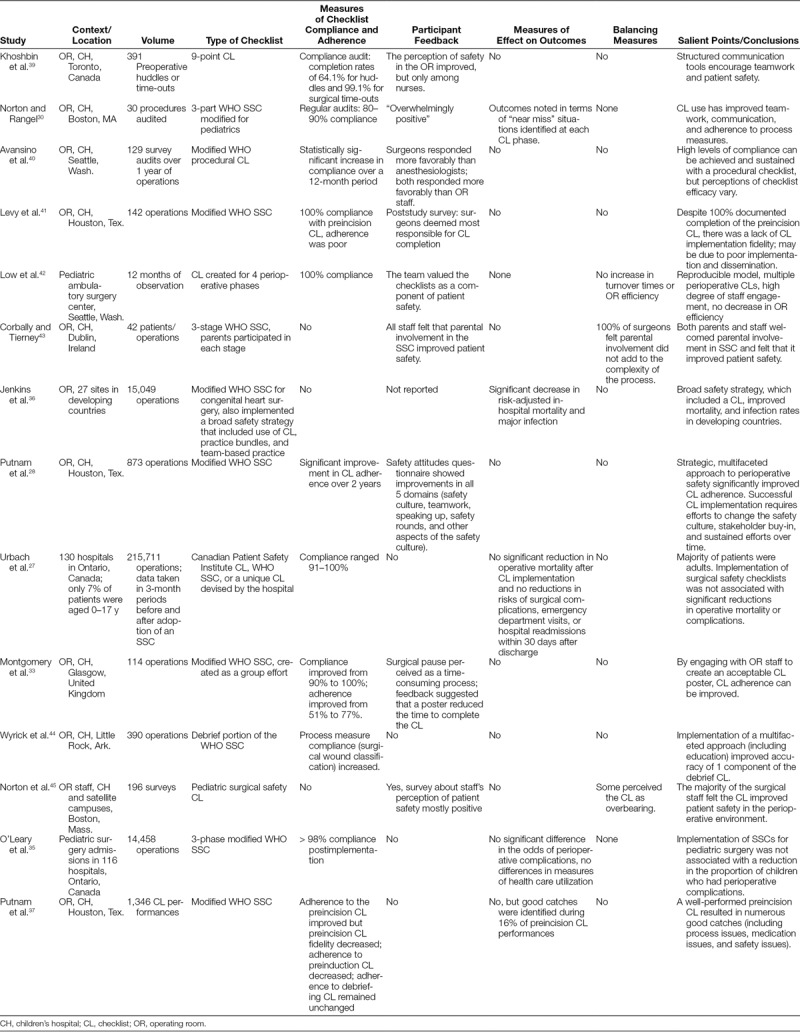
Pediatric Surgical Studies on Checklists Used in the Operating Room

### Review of the Adult Surgical Literature on Checklists

The adult literature yielded 9 systematic reviews that met the inclusion criteria, and these are reviewed here in more detail starting with oldest to newest.^11,19–26^ Unlike the majority of the pediatric surgery literature, several of these studies did evaluate the effect of checklists on morbidity and mortality. It is important to note that many of the individual studies included in each systematic review overlap those included in the other reviews, as would be expected. Therefore, there is some redundancy between systematic reviews. Table 3 (**Supplemental Digital Content**, available at ***http://links.lww.com/PQ9/A43***) shows a summary of the results.

**Table 3.**
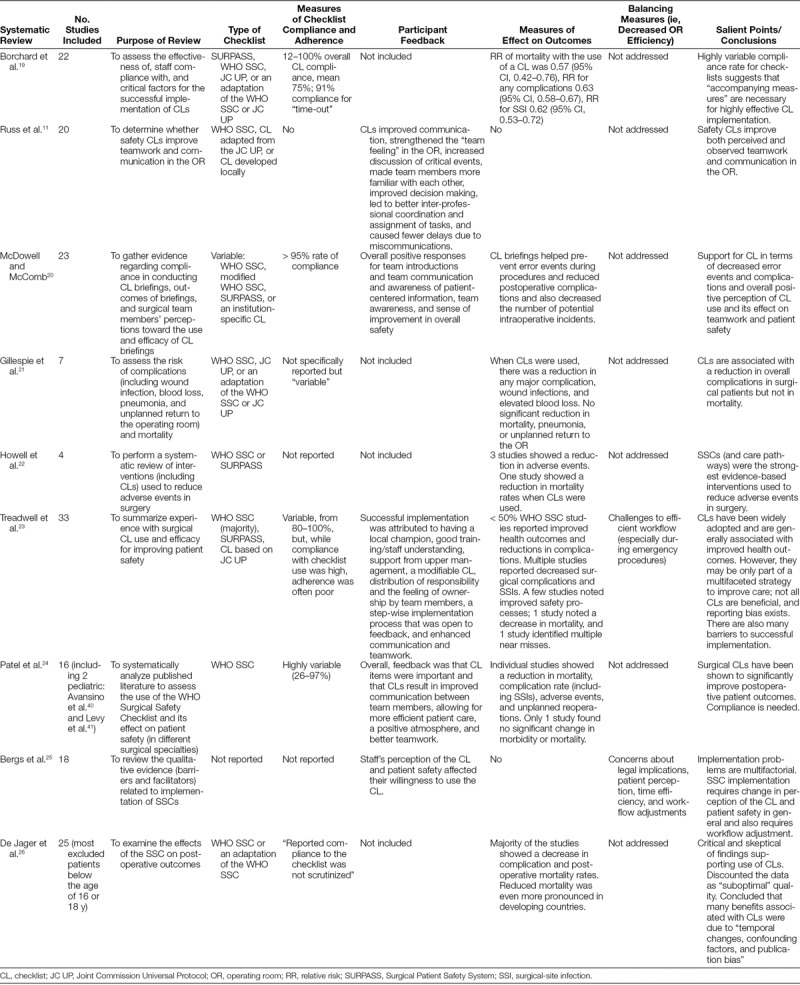
Systematic Reviews of Adult Studies on Surgical Safety Checklists

## DISCUSSION

For nearly a decade, checklist implementation has been a surgical safety initiative around the world. Since the landmark 2009 *NEJM* report showing a decrease in deaths and surgical complications after the implementation of the WHO’s SSC in 8 different hospitals,^4^ numerous studies evaluate the role of checklists in surgery. The NEJM report by Haynes et al.^4^ has been challenged, and some studies show that SSCs cause no reduction in morbidity and/or mortality.^21,27^ However, the majority of studies show that checklists improve patient outcomes, teamwork, and communication. This national survey of APSA members shows that pediatric surgeons are skeptical about the value of SSCs. The current investigators sought to understand why such skepticism exists and how to make the SSC a more meaningful safety tool for their pediatric patients.

In the current survey, the authors found that the majority of pediatric surgeons believe that checklists improve operating room communication. Both the pediatric and adult literature support this notion; checklists are one tool used to facilitate better communication in surgical settings and are shown to improve operating room teamwork.^11,28^ In 1 study of all root cause analyses submitted to the Joint Commission on Accreditation of Healthcare Organizations, communication is the most common root cause of sentinel event wrong-site surgeries.^29^ Ideally, good communication is efficient. In the current survey, several pediatric surgeons express opinions that the checklist is too long or contained elements that are irrelevant to a particular case. A checklist that contains too many elements may be thought to inhibit effective communication. As 1 pediatric surgeon said, “Like any checklist, it is used with varying degrees of a mechanical/begrudging fashion.” Acceptance of the checklist as an important safety tool means realizing that not all checklist items will benefit every patient, but all patients will benefit from some portion of the checklist. Customizing the checklist to pediatric general surgical patients should be a goal, but maintaining a routine set of items ensures that communication is standardized.

Across several studies in both the adult and pediatric literature, staff perception is noted to play a large role in the acceptance and successful adoption of checklists. To maximize its safety benefit, those people designing and using the checklist need to be engaged in the process. Some pediatric surgeons surveyed express support for the checklist and it is becoming “hard-wired” as an expected part of surgery. Perceived ownership of the checklist varies. Having an organizational culture of safety that begins with executive leadership can help foster an environment where the introduction of a checklist is well received. The onus for completing a checklist may be driven by hospital administration to satisfy a Joint Commission requirement but, ideally, is most closely overseen by leadership within the operating room who may be more “in touch” with the day-to-day use of the checklist. The investigators did note that being in a safety position is the only surgeon characteristic that correlates with believing that checklists improve patient safety or wanting the checklist used in one’s own child’s operation. As the systematic study by Berg et al.^25^ states, “The motivation for implementing an SSC differs between health care providers and hospital management.” In the Boston Children’s study, feedback from surgeons, anesthesiologists, and nursing staff is “overwhelmingly positive,”^30^ and this may have been due to participant engagement in the initiation of the checklist. After a first pilot test, feedback resulted in changes to improve the second version. Participant involvement in the creation and modification of the checklist can be beneficial but is not enough. Implementation of the checklist needs to be done thoughtfully. Repeatedly, it is seen that effective implementation strategies incorporate education and in-service training that addresses the concerns expressed by staff.^25,30^ As many studies have shown, when implemented well, checklists improve the safety culture.^23,25,31,32^

Clearly, there is a difference between checklist compliance (participants did the checklist), adherence (participants did all the parts of the checklist as it was designed), and fidelity (participants did the checklist with intent and meaning). Multiple studies show that checklist adherence is lower than compliance.^23,33^ In the pediatric literature, SSC compliance has been good in most studies. Not all elements of the checklist are being done routinely, however, perhaps because the checklist is too lengthy. The current study found that pediatric surgeons surveyed do use SSCs regularly, but that compliance is best with the preinduction and preincision, and that the postoperative debriefing checklist is much less common. It would be interesting to know why this is the case among the surgeons surveyed. It may be easier to overlook a less formal postoperative debriefing than a more formal time-out. It may be because the lead surgeon or the lead anesthesiologist or both are not always both present at the end of the case to lead the debriefing. It may be due to a lack of institutional awareness of the potential benefits of the debriefing.^34^ The relevance of the postoperative debriefing may also be a factor. If the debriefing contains elements that pertain to the transition of care of the patient leaving the operating room, it represents a key moment of team communication. However, if it contains only a review of wound classification and name of the case, it may be less valued as a component of patient safety. Better education on the benefits of doing all 3 parts of the SSC as it is designed is needed to make the debriefing an essential part for pediatric surgeons.

In the current survey, the authors found that pediatric surgeons express concern about the checklist being done in a mindlessly. When the operating room team does not embrace checklists or uses them improperly, they can have a negative effect on team dynamics.^11^ When used properly, checklists strengthen the “team feeling” in the operating room, increase discussion of critical events, improve decision making, and lead to better inter-professional coordination and assignment of tasks.^11^ One theme noted in the comments is the concern that the mindless use of the checklist can distract from thoughtful patient care. “Going through the motions” and not paying attention to the components of the checklist can detract from its utility and can contribute to surgeons’ skepticism about its benefit. By contrast, thoughtful use of the checklist can result in “good catches” or “near misses,” which will ultimately improve patient safety.

This survey shows that pediatric surgeons question the checklist’s ability to prevent adverse events. Although the majority of the pediatric literature on checklists does not address effects on complications and mortality, the adult literature shows that checklists reduce both.^19–26^ A recent retrospective study from Ontario showed no reduction in perioperative complications when SSCs were used in pediatric patients.^35^ In one of the other few studies that address outcomes in pediatric surgical patients, there is no significant decrease in morbidity or mortality, except in developing countries when a broad safety strategy that includes practice bundles and team-based practice is employed.^36^ In the pediatric population, perhaps a better measure of outcome success would be to look at the ability of the checklist to prevent errors and identify “near misses” or “good catches.” As suggested by Putnam et al.,^37^ meaningful use or good catches may be a more appropriate metric for checklist effectiveness. Wrong-site surgery is unacceptable but exceedingly rare. Still, current site-verification protocols could have prevented only two-thirds of the wrong-site surgery examined cases. Many protocols involve considerable redundancy without clear added benefit.^38^ A preincision checklist that includes site verification and pertinent radiological imaging, and has engaged participants, is a more efficient way of confirming the correct surgical site. Other good catches including identification of medication issues and allergies and confirmation of the availability of essential implants or blood products can have safety implications for a pediatric patient. As 1 pediatric surgeon responded to the survey, “SSC has definitely improved OR safety. NO wrong side procedures, NO wrong procedures since it was introduced and adopted. Also caught several ‘near misses,’ also improved timeliness of preoperative antibiotics.” The effect of the SSC can be measured by its ability to prevent harm before it happens.

This study has several limitations. The survey questions were selected based on what the APSA Quality and Safety Committee subcommittee deemed important to evaluate checklists in pediatric surgery. The response rate was only 38%, which may reflect a lack of interest in or support for checklist use among the APSA membership. The majority of respondents reported holding leadership positions at their institutions, and those in leadership positions may be more likely to support a checklist because of the belief that it improves the safety culture. In general, those who responded to the survey are more likely to be involved in patient safety initiatives, so the survey likely overestimates pediatric surgeons’ enthusiasm for checklists.

Despite being driven by Centers for Medicare and Medicaid Services and the WHO to use them, belief in SSCs among pediatric surgeons is lacking. Checklists are often implemented as part of a multi-step process to improve care. Studies show that safety attitudes and staff engagement affect successful implementation, adherence, and fidelity of the SSC. The current survey results support this. Checklists work, but only if surgeons believe in them and are engaged. Those surgeons who believe in the safety culture understand why SSCs are important. Clearly, there needs to be more education to get pediatric surgeons on board with checklists and to get them involved in the development and implementation of checklists for the pediatric surgical patients. Their “buy-in” is essential to their effective use, and the current survey shows that an alarming number of pediatric surgeons have not “bought in.” In the pediatric setting, investigators should judge the effectiveness of a checklist on its ability to foster teamwork, good communication, and improve the safety culture. With pediatric patients, the SSC can certainly prevent errors, but its benefit may not be in its direct effect on patient outcomes. When compared with adults, the pediatric adverse and “never” event rates are very low. Therefore, using complications and mortality rates as a checklist metric is unrealistic. At a time when quality improvement is at the forefront of health care, and when the Children’s Surgery Verification program is beginning to set expectations for pediatric surgical care in the United States, one must look to safety initiatives that have shown promising results to be the foundation of surgical care for children. Checklists are such an initiative. One must continually engage in such safety efforts when seeking to provide better, safer care for the patient population.

## DISCLOSURE

The authors have no financial interest to declare in relation to the content of this article.

## Supplementary Material

SUPPLEMENTARY MATERIAL

## References

[1] Gawande A The Checklist Manifesto: How to Get Things Right. 2011 New York, N.Y.: Metropolitan Books of Henry Holt and Co.

[2] World Alliance for Patient Safety. Safe surgery saves lives: second global patient safety challenge. Available at http://www.who.int/patientsafety/safesurgery/knowledge_base/SSSL_Brochure_finalJun08.pdf. Accessed May 10, 2017.

[3] WHO surgical safety checklist. Available at http://www.who.int/patientsafety/safesurgery/tools_resources/SSSL_Checklist_finalJun08.pdf. Accessed May 10, 2017.

[4] Haynes AB Weiser TG Berry WR; Safe Surgery Saves Lives Study Group. A surgical safety checklist to reduce morbidity and mortality in a global population. N Engl J Med. 2009;360:491 499. 19144931 10.1056/NEJMsa0810119

[5] CMS.gov. ASC quality reporting. 2017 Available at https://www.cms.gov/Medicare/Quality-Initiatives-Patient-Assessment-Instruments/ASC-Quality-Reporting/. Accessed May 10, 2017.

[6] World Health Organization. New scientific evidence supports WHO findings: a surgical safety checklist could save hundreds of thousands of lives. 2011 Available at http://www.who.int/patientsafety/safesurgery/checklist_saves_lives/en/index.html. Accessed May 15, 2017.

[7] Takala RS Pauniaho SL Kotkansalo A A pilot study of the implementation of WHO surgical checklist in Finland: improvements in activities and communication. Acta Anaesthesiol Scand. 2011;55:1206 1214. 22092125 10.1111/j.1399-6576.2011.02525.x

[8] Helmiö P Blomgren K Takala A Towards better patient safety: WHO Surgical Safety Checklist in otorhinolaryngology. Clin Otolaryngol. 2011;36:242 247. 21481197 10.1111/j.1749-4486.2011.02315.x

[9] Böhmer AB Wappler F Tinschmann T The implementation of a perioperative checklist increases patients’ perioperative safety and staff satisfaction. Acta Anaesthesiol Scand. 2012;56:332 338. 22188135 10.1111/j.1399-6576.2011.02590.x

[10] Haynes AB Weiser TG Berry WR; Safe Surgery Saves Lives Study Group. Changes in safety attitude and relationship to decreased postoperative morbidity and mortality following implementation of a checklist-based surgical safety intervention. BMJ Qual Saf. 2011;20:102 107. 10.1136/bmjqs.2009.040022 21228082

[11] Russ S Rout S Sevdalis N Do safety checklists improve teamwork and communication in the operating room? A systematic review. Ann Surg. 2013;258:856 871. 24169160 10.1097/SLA.0000000000000206

[12] Reames BN Scally CP Thumma JR Evaluation of the effectiveness of a surgical checklist in Medicare patients. Med Care. 2015;53:87 94. 25464163 10.1097/MLR.0000000000000277 PMC4262562

[13] Singer SJ Molina G Li Z Relationship between operating room teamwork, contextual factors, and safety checklist performance. J Am Coll Surg. 2016;223:568 580.e2. 27469627 10.1016/j.jamcollsurg.2016.07.006

[14] Glaser BG Strauss AL The Discovery of Grounded Theory: Strategies for Qualitative Research. 1967 Chicago, Ill.: Aldine.

[15] Bradley EH Curry LA Devers KJ Qualitative data analysis for health services research: developing taxonomy, themes, and theory. Health Serv Res. 2007;42:1758 1772. 17286625 10.1111/j.1475-6773.2006.00684.x PMC1955280

[16] Krippendorff K Content Analysis. An Introduction to its Metho dology. The Sage Commtext Series. 1980 London, United Kingdom: Sage Publications.

[17] Macdonald AL Sevdalis N Patient safety improvement interventions in children’s surgery: a systematic review. J Pediatr Surg. 2017;52:504 511. 27717565 10.1016/j.jpedsurg.2016.09.058

[18] Lagoo J Lopushinsky SR Haynes AB Effectiveness and meaningful use of paediatric surgical safety checklists and their implementation strategies: a systematic review with narrative synthesis. BMJ Open. 2017;7:e016298. 10.1136/bmjopen-2017-016298 PMC5652514 29042377

[19] Borchard A Schwappach DL Barbir A A systematic review of the effectiveness, compliance, and critical factors for implementation of safety checklists in surgery. Ann Surg. 2012;256:925 933. 22968074 10.1097/SLA.0b013e3182682f27

[20] McDowell DS McComb SA Safety checklist briefings: a systematic review of the literature. AORN J. 2014;99:125 137.e13. 24369977 10.1016/j.aorn.2013.11.015

[21] Gillespie BM Chaboyer W Thalib L Effect of using a safety checklist on patient complications after surgery: a systematic review and meta-analysis. Anesthesiology. 2014;120:1380 1389. 24845919 10.1097/ALN.0000000000000232

[22] Howell AM Panesar SS Burns EM Reducing the burden of surgical harm: a systematic review of the interventions used to reduce adverse events in surgery. Ann Surg. 2014;259:630 641. 24368639 10.1097/SLA.0000000000000371

[23] Treadwell JR Lucas S Tsou AY Surgical checklists: a systematic review of impacts and implementation. BMJ Qual Saf. 2014;23:299 318. 10.1136/bmjqs-2012-001797 PMC3963558 23922403

[24] Patel J Ahmed K Guru KA An overview of the use and implementation of checklists in surgical specialities—a systematic review. Int J Surg. 2014;12:1317 1323. 25448652 10.1016/j.ijsu.2014.10.031

[25] Bergs J Lambrechts F Simons P Barriers and facilitators related to the implementation of surgical safety checklists: a systematic review of the qualitative evidence. BMJ Qual Saf. 2015;24:776 786. 10.1136/bmjqs-2015-004021 26199428

[26] de Jager E McKenna C Bartlett L Postoperative adverse events inconsistently improved by the World Health Organization Surgical Safety Checklist: a systematic literature review of 25 studies. World J Surg. 2016;40:1842 1858. 27125680 10.1007/s00268-016-3519-9 PMC4943979

[27] Urbach DR Govindarajan A Saskin R Introduction of surgical safety checklists in Ontario, Canada. N Engl J Med. 2014;370:1029 1038. 24620866 10.1056/NEJMsa1308261

[28] Putnam LR Levy SM Sajid M Multifaceted interventions improve adherence to the surgical checklist. Surgery. 2014;156:336 344. 24947646 10.1016/j.surg.2014.03.032

[29] Sentinel events: approaches to error reduction and prevention. Jt Comm J Qual Improv. 1998;24:175 186. 9589330 10.1016/s1070-3241(16)30370-4

[30] Norton EK Rangel SJ Implementing a pediatric surgical safety checklist in the OR and beyond. AORN J. 2010;92:61 71. 20619773 10.1016/j.aorn.2009.11.069

[31] Haugen AS Søfteland E Eide GE Impact of the World Health Organization’s Surgical Safety Checklist on safety culture in the operating theatre: a controlled intervention study. Br J Anaesth. 2013;110:807 815. 23404986 10.1093/bja/aet005 PMC3630285

[32] Lee SL The extended surgical time-out: does it improve quality and prevent wrong-site surgery? Perm J. 2010;14:19 23. 20740127 10.7812/tpp/09-124 PMC2912716

[33] Montgomery K Khan I Thomson K Improving the practice of the World Health Organisation’s surgical pause checklist at a tertiary paediatric surgical unit. Scott Med J. 2016;61:88 91. 27288187 10.1177/0036933016649873

[34] Bartz-Kurycki MA Anderson KT Abraham JE Debriefing: the forgotten phase of the surgical safety checklist. J Surg Res. 2017;213:222 227. 28601318 10.1016/j.jss.2017.02.072

[35] O’Leary JD Wijeysundera DN Crawford MW Effect of surgical safety checklists on pediatric surgical complications in Ontario. CMAJ. 2016;188:E191 E198. 26976960 10.1503/cmaj.151333 PMC4902710

[36] Jenkins KJ Castañeda AR Cherian KM Reducing mortality and infections after congenital heart surgery in the developing world. Pediatrics. 2014;134:e1422 e1430. 25311607 10.1542/peds.2014-0356

[37] Putnam LR Anderson KT Diffley MB Meaningful use and good catches: more appropriate metrics for checklist effectiveness. Surgery. 2016;160:1675 1681. 27473370 10.1016/j.surg.2016.04.038

[38] Kwaan MR Studdert DM Zinner MJ Incidence, patterns, and prevention of wrong-site surgery. Arch Surg. 2006;141:353 357; discussion 357. 16618892 10.1001/archsurg.141.4.353

[39] Khoshbin A Lingard L Wright JG Evaluation of preoperative and perioperative operating room briefings at the Hospital for Sick Children. Can J Surg. 2009;52:309 315. 19680516 PMC2724800

[40] Avansino JR Javid P Katz C Implementation of a standardized procedural checklist in a children’s hospital. Am J Surg. 2011;201:660 665. 21545918 10.1016/j.amjsurg.2011.01.014

[41] Levy SM Senter CE Hawkins RB Implementing a surgical checklist: more than checking a box. Surgery. 2012;152:331 336. 22770952 10.1016/j.surg.2012.05.034

[42] Low DK Reed MA Geiduschek JM Striving for a zero-error patient surgical journey through adoption of aviation-style challenge and response flow checklists: a quality improvement project. Paediatr Anaesth. 2013;23:571 578. 23373830 10.1111/pan.12121

[43] Corbally MT Tierney E Parental involvement in the preoperative surgical safety checklist is welcomed by both parents and staff. Int J Pediatr. 2014;2014:791490. 24834075 10.1155/2014/791490 PMC4009146

[44] Wyrick DL Smith SD Dassinger MS Implementation of the World Health Organization checklist and debriefing improves accuracy of surgical wound class documentation. Am J Surg. 2015;210:1051 1054; discussion 1054. 26460055 10.1016/j.amjsurg.2015.08.010

[45] Norton EK Singer SJ Sparks W Operating room clinicians’ attitudes and perceptions of a pediatric surgical safety checklist at 1 institution. J Patient Saf. 2016;12:44 50. 25010191 10.1097/PTS.0000000000000120

