# Supplementary material for: Surgical Safety Checklists in Children’s Surgery: Surgeons’ Attitudes and Review of the Literature
Source: Pediatr Qual Saf. 2018 Oct 16;3(5):e108. doi: 10.1097/pq9.0000000000000108 (PMC6221594; doi:10.1097/pq9.0000000000000108)
Supplement: SUPPLEMENTARY MATERIAL [file pqs-3-e108-s001.docx]

**Table 3:** **Systematic reviews of adult studies on surgical safety checklists**

| **Systematic review** | **Number of studies included** | **Purpose of review** | **Type of checklist** | **Measures of checklist compliance and adherence** | **Participant feedback** | **Measures of effect on outcomes** | **Balancing measures (i.e., decreased OR efficiency)** | **Salient points/**  **conclusions** |
| --- | --- | --- | --- | --- | --- | --- | --- | --- |
| Borchard et al. 2012^33^ | 22 | To assess the effectiveness of, staff compliance with, and critical factors for the successful implementation of CLs | SURPASS, WHO SSC, JC UP, or an adaptation of the WHO SSC or JC UP | 12%-100% overall CL compliance, mean 75%; 91% compliance for “time-out” | Not included | RR of mortality with the use of a CL was 0.57 (95% CI: 0.42-0.76), RR for any complications 0.63 (95% CI: 0.58-0.67), RR for SSI 0.62 (95% CI: 0.53-0.72) | Not addressed | Highly variable compliance rate for checklists suggests that “accompanying measures” are necessary for highly effective CL implementation. |
| Russ et al. 2013^11^ | 20 | To determine whether safety CLs improve teamwork and communication in the OR | WHO SSC, CL adapted from the JC UP, or CL developed locally | No | CLs improved communication, strengthened the “team feeling” in the OR, increased discussion of critical events, made team members more familiar with each other, improved decision making, led to better inter-professional coordination and assignment of tasks, and caused fewer delays due to miscommunications. | No | Not addressed | Safety CLs improve both perceived and observed teamwork and communication in the OR. |
| McDowell et al. 2014^34^ | 23 | To gather evidence regarding compliance in conducting CL briefings, outcomes of briefings, and surgical team members’ perceptions toward the use and efficacy of CL briefings | Variable: WHO SSC, modified WHO SSC, SURPASS, or an institution-specific CL | >95% rate of compliance | Overall positive responses for team introductions and team communication and awareness of patient-centered information, team awareness, and sense of improvement in overall safety | CL briefings helped prevent error events during procedures and reduced postoperative complications and also decreased the number of potential intraoperative incidents. | Not addressed | Support for CL in terms of decreased error events and complications and overall positive perception of CL use and its effect on teamwork and patient safety |
| Gillespie et al. 2014^35^ | 7 | To assess the risk of complications (including wound infection, blood loss, pneumonia, and unplanned return to the operating room) and mortality | WHO SSC, JC UP, or an adaptation of the WHO SSC or JC UP | Not specifically reported but “variable” | Not included | When CLs were used, there was a reduction in any major complication, wound infections, and elevated blood loss. No significant reduction in mortality, pneumonia, or unplanned return to the OR | Not addressed | CLs are associated with a reduction in overall complications in surgical patients but not in mortality. |
| Howell et al. 2014^36^ | 4 | To perform a systematic review of interventions (including CLs) used to reduce adverse events in surgery | WHO SSC or SURPASS | Not reported | Not included | 3 studies showed a reduction in adverse events. One study showed a reduction in mortality rates when CLs were used. | Not addressed | SSCs (and care pathways) were the strongest evidence-based interventions used to reduce adverse events in surgery. |
| Treadwell et al. 2014^37^ | 33 | To summarize experience with surgical CL use and efficacy for improving patient safety | WHO SSC (majority), SURPASS, CL based on JC UP | Variable, from 80%-100%, but, while compliance with checklist use was high, adherence was often poor | Successful implementation was attributed to having a local champion, good training/staff understanding, support from upper management, a modifiable CL, distribution of responsibility and the feeling of ownership by team members, a step-wise implementation process that was open to feedback, and enhanced communication and teamwork. | <50% WHO SSC studies reported improved health outcomes and reductions in complications. Multiple studies reported decreased surgical complications and SSIs. A few studies noted improved safety processes; one study noted a decrease in mortality, and one study identified multiple near misses. | Challenges to efficient workflow (especially during emergency procedures) | CLs have been widely adopted and are generally associated with improved health outcomes. However, they may be only part of a multifaceted strategy to improve care; not all CLs are beneficial, and reporting bias exists. There are also many barriers to successful implementation. |
| Patel et al. 2014^38^ | 16  (including 2 pediatric: Avansino et al. and Levy et al.) | To systematically analyze published literature to assess the use of the WHO Surgical Safety Checklist and its effect on patient safety (in different surgical specialties) | WHO SSC | Highly variable (26%-97%) | Overall, feedback was that CL items were important and that CLs result in improved communication between team members, allowing for more efficient patient care, a positive atmosphere, and better teamwork. | Individual studies showed a reduction in mortality, complication rate (including SSIs), adverse events, and unplanned reoperations. Only one study found no significant change in morbidity or mortality. | Not addressed | Surgical CLs have been shown to significantly improve postoperative patient outcomes. Compliance is needed. |
| Bergs et al. 2014^39^ | 18 | To review the qualitative evidence (barriers and facilitators) related to implementation of SSCs | Not reported | Not reported | Staff’s perception of the CL and patient safety affected their willingness to use the CL. | No | Concerns about legal implications, patient perception, time efficiency, and workflow adjustments | Implementation problems are multifactorial. SSC implementation requires change in perception of the CL and patient safety in general and also requires workflow adjustment. |
| De Jager et al. 2016^40^ | 25  (most excluded patients below the age of 16 or 18) | To examine the effects of the SSC on postoperative outcomes | WHO SSC or an adaptation of the WHO SSC | “Reported compliance to the checklist was not scrutinized” | Not included | Majority of the studies showed a decrease in complication and postoperative mortality rates. Reduced mortality was even more pronounced in developing countries. | Not addressed | Critical and skeptical of findings supporting use of CLs. Discounted the data as “sub-optimal” quality. Concluded that many benefits associated with CLs were due to “temporal changes, confounding factors and publication bias” |

OR, operating room; CL, checklist; SURPASS, Surgical Patient Safety System; WHO, World Health Organization; SSC, Surgical Safety Checklist; JC UP, Joint Commission Universal Protocol; RR, relative risk; SSI, surgical site infection
